# Comorbidities, sequelae, blood biomarkers and their associated clinical outcomes in the Mount Sinai Health System COVID-19 patients

**DOI:** 10.1371/journal.pone.0253660

**Published:** 2021-07-06

**Authors:** Agnieszka Brojakowska, Abrisham Eskandari, Malik Bisserier, Jeffrey Bander, Venkata Naga Srikanth Garikipati, Lahouaria Hadri, David A. Goukassian, Kenneth M. Fish

**Affiliations:** 1 Cardiovascular Research Institute, Icahn School of Medicine at Mount Sinai, New York, New York, United States of America; 2 Department of Emergency Medicine, Ohio State University Wexner Medical Center, Columbus, Ohio, United States of America; Azienda Ospedaliero Universitaria Careggi, ITALY

## Abstract

With the continuing rise of SARS-CoV2 infection globally and the emergence of various waves in different countries, understanding characteristics of susceptibility to infection, clinical severity, and outcomes remain vital. In this retrospective study, data was extracted for 39,539 patients from the de-identified Mount Sinai Health System COVID-19 database. We assessed the risk of mortality based on the presence of comorbidities and organ-specific sequelae in 7,032 CoV2 positive (+) patients. Prevalence of cardiovascular and metabolic comorbidities was high among SARS-CoV2+ individuals. Diabetes, obesity, coronary artery disease, hypertension, atrial fibrillation, and heart failure all increased overall mortality risk, while asthma did not. Ethnicity modified the risk of mortality associated with these comorbidities. With regards to secondary complications in the setting of infection, individuals with acute kidney injury and acute myocardial injury showed an increase in mortality risk. Cerebral infarcts and acute venous thromboembolic events were not associated with increased risk of mortality. Biomarkers for cardiovascular injury, coagulation, and inflammation were compared between deceased and survived individuals. We found that cardiac and coagulation biomarkers were elevated and fell beyond normal range more often in deceased patients. Several, but not all, inflammatory markers evaluated were increased in deceased patients. In summary, we identified comorbidities and sequelae along with peripheral blood biomarkers that were associated with elevated clinical severity and poor outcomes in COVID-19 patients. Overall, these findings detail the granularity of previously reported factors which may impact susceptibility, clinical severity, and mortality during the course of COVID-19 disease.

## Introduction

During the global COVID-19 pandemic, caused by the severe acute respiratory syndrome Coronavirus 2 (SARS-CoV2), the World Health Organization has reported more than 166 million confirmed cases of COVID-19 worldwide and 3,459,996 deaths as of May 24th 2021. Early manifestation of disease is associated with respiratory tract infections causing flu-like symptoms, which in some individuals may develop into acute respiratory distress syndrome (ARDS) and acute respiratory failure [1–3]. Although most patients present with mild symptoms and good prognosis, some develop severe cardiovascular complications such as coagulopathy, heart failure, acute coronary syndrome, and coronary artery aneurysm [4]. Despite significant advances made since its emergence, the pathogenesis and clinical features of COVID-19 and its clinical course remain largely poorly understood. Importantly, there is no proven effective therapeutics available for the COVID-19 treatment. As part of efforts to control the adverse effects of COVID-19 disease, numerous studies attempted to identify demographic modifiers and comorbidities associated with COVID-19 susceptibility and mortality using limited patient cohorts [2, 4–9].

Many studies have highlighted the prevalence of pre-existing cardiovascular, metabolic, renal, and pulmonary diseases in patients with more severe cases [10], along with increased risk of mortality [11]. Multiorgan dysfunction, primarily involving the heart, kidneys, lungs, and liver, has also been noted [12, 13]. Elevated cardiac, clotting and inflammatory biomarkers have been observed [14–16]. However, many of these studies have limited cohorts, were conducted on inpatient populations, and were selective of severe cases in assessing the associated outcomes following SARS-CoV2 infection [2, 4–9].

The role of underlying comorbidities on susceptibility to SARS-CoV-2 infection and the clinical severity of COVID-19 remains unclear. Given the renewed increase in global incidence of COVID-19, defining the characteristics that impact susceptibility to SARS-CoV-2 infection, disease progression, and outcomes remains of great importance. A better understanding of the evolving epidemiology and clinical spectrum is vital to provide comprehensive guidance with planning, prioritizing healthcare system resources, and accessibility to appropriate therapies and follow-up strategies. Our study included outpatients and patients that presented at the emergency departments throughout the Mount Sinai Hospital System (MSHS) throughout the New York City area. Therefore, our study presumably includes less severe cases that did not require hospitalization, and therefore is reflective of the wide clinical course of the disease.

In this study, we aimed to assess mortality in COVID-19 patients with pre-existing comorbidities and sequelae using one of the largest de-identified databases. The MSHS COVID-19 database contains records of 7,032 SARS-CoV-2 positive patients (CoV2+) who entered the health system from 02/28/2020 to 06/08/2020. We further sought to understand whether the levels of serum biomarkers for cardiovascular injury, coagulation, and inflammation were associated with clinical outcomes.

## Materials and methods

### Electronic medical record data mining

**Fig 1:**
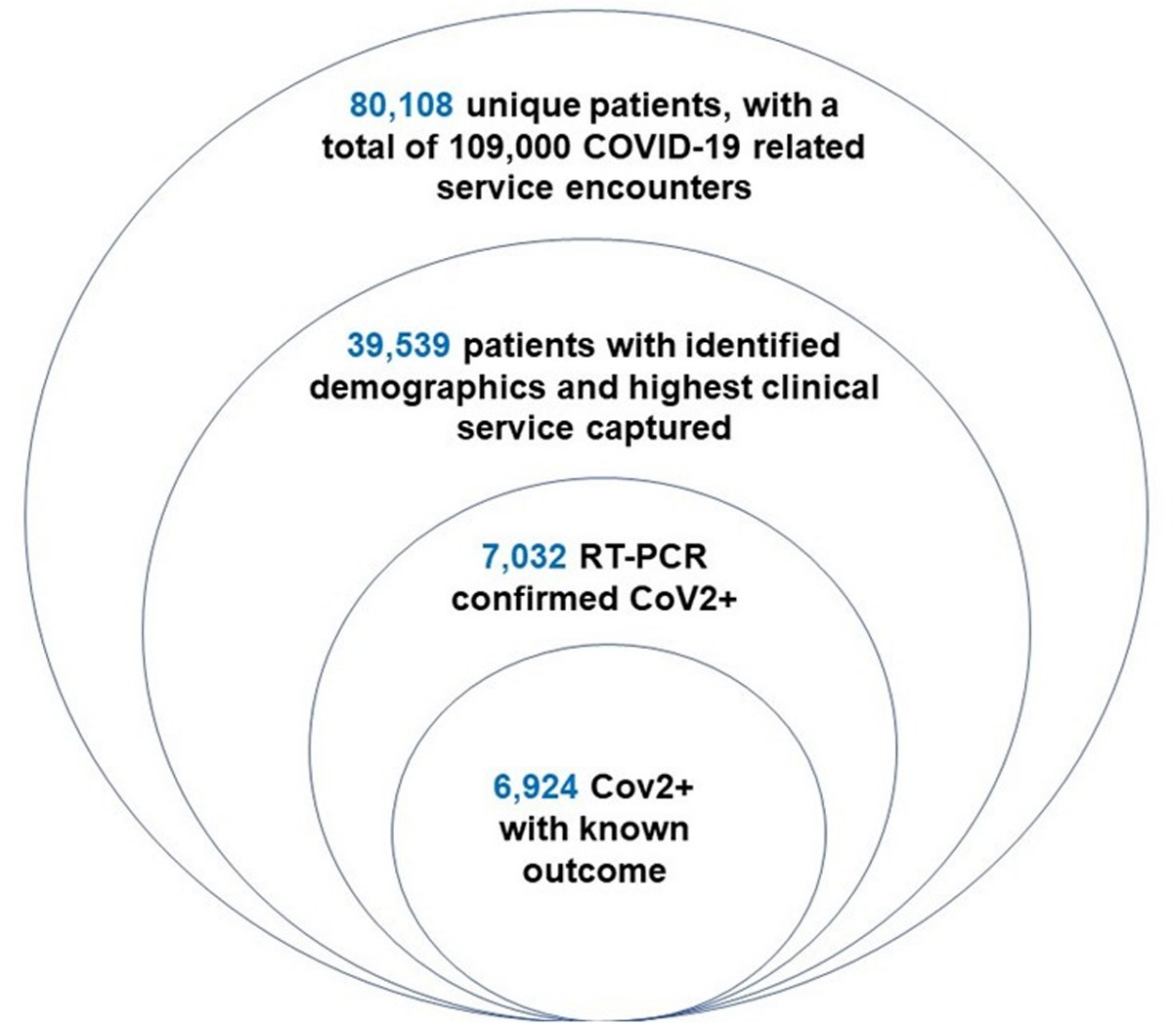
Subgroup breakdown for analyses. From February 28th to June 8th, 2020, this database consisted of 80,108 masked electronic record numbers (representing individual patients) that entered the MSHS with a COVID-19 related encounter, including 109,000 individual service encounters. We analyzed de-identified patient data from 39,625 patients in the Mount Sinai Health System (MSHS), which had recorded metrics with respect to sex, age, and race. We found that 6,537 of them were determined to be CoV2+ by nasopharyngeal qRT-PCR confirmed tests, while 495 patients were presumed positive for a total of 7,032 COVID-19 cases. We excluded all patients for which an outcome was not yet known, limiting the analysis to a total of 6,924 patients—5,629 individuals who survived and 1,295 deceased.

The MSHS, with hospitals and outpatient sites throughout the metropolitan NYC area, was at the epicenter of the global COVID-19 pandemic. A de-identified MSHS COVID-19 patient database was generated from the electronic medical record (EMR; EPIC) system and made available to the MSHS research community. From February 28th to June 8th, 2020, this database consisted of 80,108 masked electronic record numbers (representing individual patients) that entered the MSHS with a COVID-19 related encounter, including 109,000 individual service encounters including outpatient (OP) visits which entail telehealth and urgent care visits, emergency department (ED) visits, and inpatient (IP) admissions. IP admissions were further categorized into non-ICU or ICU admission. For the remainder of this discussion, non-ICU patients will be referred to as IP. Our patient filtration process is outlined in Fig 1.

Multiple variables were featured in the database and used in stratification and characterization of the cohort’s associated COVID-19 risk. These included age, sex, race, ethnicity, SARS-CoV2 qRT-PCR testing, encounter types, location of care, admission type codes, discharge locations, deceased indicators, comorbidities, sequelae, and varying biomarkers. Consistent with the database, only ethnic groups represented in the US Census were included in our analyses: White, Hispanic, Black or African American, Other, Asian, Native Hawaiian or Pacific Islander, and American Indian or Alaskan native. Black or African Americans will be referred to as Black for the remainder of the manuscript. A combined race and ethnicity variable defined by Mount Sinai’s COVID Disparities and Equity Taskforce was used to stratify demographic information to better capture Hispanic/Latinx patients. Considering this combined variable, we will refer to race and ethnicity simply as ethnicity for the remainder of the manuscript.

#### Data preparation: COVID-19 severity score

To reflect COVID-19 infection severity, we created a scoring system based on the patient’s clinical service encounter such that OP visits represented mild cases, and the degree of severity subsequently increased from ED visits to IP admissions and finally to ICU admissions, which represented the most severe clinical course. Unspecified service encounters, including radio-oncology and neuropsychiatric visits (n = 67 patients), were not included. After a severity score was calculated for each encounter, only the first encounter with the maximum severity score was selected to represent an individual patient’s clinical course. This scoring system limited the patient’s representation in our studied cohort to only one encounter and allowed for a more accurate representation of a patient’s clinical course. We excluded encounters that included unspecified/un-identified data with respect to age, sex, and ethnicity. This filtration, in addition to the removal of patients with unidentified service, encounters limited our analysis to include 39,539 patients.

#### SARS CoV-2 qRT-PCR testing

With regards to COVID-19 nasopharyngeal qRT-PCR testing status, the database included individuals who tested positive (CoV2+), presumed positive, negative (COV2-), and individuals who were not tested. Presumed positive indicates that a patient’s positive qRT-PCR result has not yet been confirmed by the Center for Disease Control and Prevention (CDC). Within our total cohort of 39,539 patients, 17.19% were CoV2+ (which includes presumed positives), 49.42% were CoV2-, and 32.75% were not tested for the SARS-CoV2 antigen. For our analyses we focused on patients with positive (n = 6,537 patients) or presumed positive (n = 495 patients) qRT-PCR results (i.e., CoV2+ patients). Additionally, we excluded all patients for which an outcome was not yet known, limiting the analysis to a total of 6,924 patients—5,629 individuals who survived and 1,295 deceased. The count of survivors versus deceased individuals is reflective of results as of June 8^th^ 2020. It is possible that discharge proceeds the event of death or death occurs at home for outpatients. Considering our calculations were performed on the basis of COVID-19 survivors and deceased populations, we further excluded patients with unknown outcomes, i.e., were not discharged or deceased. Considering 93.36% of our deceased patient population is captured in the IP and ICU units, we removed patients whose discharge location was 1) unknown ("blank", n = 101) or 2) involved "admission to inpatient" (n = 7). The latter category represented ED patients with a single encounter at MSHS that did not end up being admitted to IP for unclear reasons. Of all patients discharged to hospice (n = 45), 18 were indicated as deceased, and 27 were indicated as survivors. Despite the poor prognosis of hospice patients, we included these patients in our analysis to avoid an assumption on the outcome—they represent 0.48% of all survivors.

### Risk stratifying mortality based on comorbidities and COVID-19 sequelae

We calculated the relative risk (RR) of mortality among our deceased patient population with known cardiovascular, pulmonary, renal, and metabolic comorbidities as well as in individuals who developed clinical complications secondary to COVID-19 infection. All comorbidities and sequelae were active issues on a patient’s problem list at the time of the given encounter. Comorbidities considered included metabolic [n = 2,495, diabetes (DM) and obesity (BMI > 30kg/m^2^)], cardiovascular [n = 2,010, coronary artery disease (CAD), hypertension (HTN), atrial fibrillation (A Fib), and heart failure (HF)], renal [n = 572, chronic kidney disease (CKD)], and pulmonary [n = 447, asthma and chronic obstructive pulmonary disease (COPD)] categories. Sequelae considered included renal [n = 78, acute kidney injury (AKI)], cardiovascular [n = 109, acute myocardial infarction (AMI) and acute venous thromboembolism (AVTE)], cerebrovascular [n = 46, cerebral infarction (CVA) and intracerebral hemorrhage (ICH)], and pulmonary [n = 29, acute respiratory distress syndrome (ARDS)].

### Examination of biomarkers in peripheral blood

We analyzed variance in different lab values between survivors and deceased individuals, including cardiovascular biomarkers [troponin I (TnI) and brain natriuretic peptide (BNP)], markers of inflammation [IL-8, IL-1b, IL-6, TNFα, C reactive protein (CRP)] as well as markers of coagulation [platelets, mean platelet volume (MPV), fibrinogen, d-dimer, and active partial prothrombin time (aPTT)]. The type and frequency of lab results vary per individual. Markers may be missing for certain patients. Results were considered for all individuals with at least one recorded lab result for the marker of interest; average value across all lab results was used.

### Statistical analysis

All statistical analyses were conducted using GraphPad Prism version 7.00 for Windows. Relative risk comparison for each comorbidity and sequela was done independently. For example, to assess the relative risk of mortality in patients with CAD, the total CoV2+ population was considered. Within this population, relative risk analysis was done to compare the mortality risk of patients with CAD and those without CAD. The mortality risk of patients with CAD is the number of SARS-CoV2+ patients with CAD who were deceased divided by the number of total SARS-CoV2+ patients with CAD. The mortality risk of patients without CAD is the number of SARS-CoV2+ patients without CAD who were deceased divided by the number of total SARS-CoV2+ patients without CAD. The former mortality risk was then divided by the latter relative risk to obtain the relative risk values. This process was repeated for each comorbidity and each sequela. However, it is possible that these individuals have one or more other comorbidities and/or sequela and therefore were represented in multiple analyses. The H_0_ for relative risk analysis states RR = 1.0; neither group faces a greater risk. The Fisher’s exact test was used for the computation of the p-value. Two-sided p-value calculations were done at a 95% CI (α = 0.05) by the Koopman asymptomatic score method.

We compared the mean of average lab results in survivors versus deceased individuals for a given biomarker of interest using unpaired two-tailed unpaired t-test with Welch’s correction at 95% CI, where H_0_ states that there is no statistically significant different in means between the compared groups. ROUT method was used to identify outliers where definitive outliers were removed (Q = 0.1%). The calculations were done at a 95% CI (α = 0.05). Corrections for multiple hypothesis testing were not done due to its unavailability on the GraphPad Prism software used. However, per the Bonferroni correction, the α value could be adjusted by dividing the α value (α = 0.05) by the number of comparisons (n = 12). The new α value would be 0.004. All p values calculated still fall below this threshold and would therefore still be significant.

## Results

### Ethnicity

We characterized the patient population based on their ethnicity. The CoV2+ patient population (n = 7,032) is represented by 27.3% Hispanics, 28.1% Blacks or African Americans, 25.9% Whites, 13.9% Others, and 4.6% Asians. Note, here and elsewhere in the manuscript, Blacks or African Americans will be referred to as Blacks.

### Comorbidities and mortality

The percentage of the CoV2+ population with at least one comorbidity from the corresponding categories are—35.6% metabolic, 28.6% cardiovascular, 8.1% renal, and 6.4% pulmonary (Table 1A). Within these categories, obesity was most prevalent, followed by hypertension (HTN), diabetes mellitus (DM), coronary artery disease (CAD), chronic kidney disease (CKD), heart failure (HF), atrial fibrillation (AFib), asthma, and lastly chronic obstructive pulmonary disease (COPD) (Table 1B). Of note, an individual may be represented in several categories depending on their number of pre-existing conditions. Mechanisms by which these comorbidities may affect the outcome of COVID-19 disease is unclear. Nonetheless, comorbidities could modify risks of infection, severity of COVID-19 clinical course, and response to administered therapies.

**Table 1:** Overall prevalence of comorbidities.

**A.**	
**Comorbidity Categories**	**Percent**
Metabolic	35.6
Cardiovascular	28.6
Renal	8.1
Pulmonary	6.4
**B.**	
**Comorbidity**	**Absolute Number (%)**
Obesity (BMI>30)	1,805 (26.1)
Hypertension (HTN)	1,681 (24.3)
Diabetes (DM)	1,067 (15.4)
Coronary Artery Disease (CAD)	592 (8.6)
Chronic Kidney Disease (CKD)	558 (8.1)
Heart Failure (HF)	349 (5.0)
Atrial Fibrillation (A Fib)	299 (4.3)
Asthma	288 (4.2)
Chronic Obstructive Pulmonary Disease (COPD)	182 (2.6)

(A) Of the 7,032 patients who presented to the Mount Sinai Health System with confirmed or presumed COVID-19 disease and known discharge/deceased status, we assessed the prevalence of having at least one metabolic, cardiovascular, renal, and pulmonary comorbidity. (B) The breakdown of prevalence by all specific comorbidities considered is shown. Among this population, there is a high prevalence of metabolic comorbidities, including obesity (defined as a BMI of greater than 30 kg/m^2^) and diabetes, followed by cardiovascular comorbidities, with a particular emphasis on the prevalence of hypertension. Of note, patients may be represented in more than one group based on their underlying medical history.

**Fig 2:**
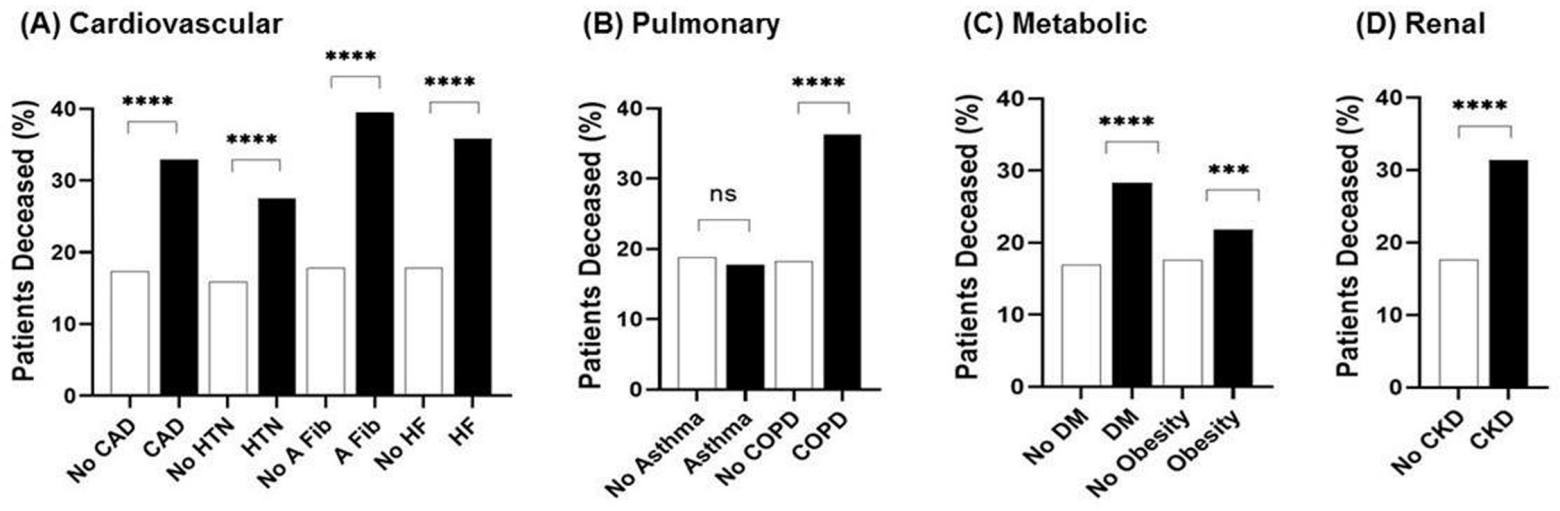
Relative risk of mortality from COVID-19 in individuals with underlying comorbidities across all ages, sexes, and ethnicities. Of the 7,032 patients who presented to the Mount Sinai Health System with confirmed or presumed COVID-19 disease and known discharge/deceased status, the relative risk of mortality from COVID-19 disease is higher in individuals with a specific (A) cardiovascular, (B) pulmonary, (C) metabolic, or (D) renal comorbidity compared to their deceased reference group which did not have the given comorbidity. Interestingly, patients with asthma (B) are the only population with no significant risk of mortality rate compared to their reference group (p = 0.70). ****p < 0.0001, ***p <0.001, **p <0.01, *p <0.05, ns = not significant.

All evaluated comorbidities, with the exception of asthma, were associated with increased risk of mortality (Fig 2A–2D). Relative risks are reported here along with the confidence intervals (RR, [CI]). AFib modified mortality risk more than other cardiovascular comorbidities (RR = 2.22, [1.90, 2.57]), followed by HF (RR = 2.201, [1.73, 2.33]), CAD (RR = 1.90, [1.67, 2.15]), and HTN (RR = 1.73, [1.57, 1.91]) (Fig 2A). Within pulmonary comorbidities, COPD increased mortality risk 2-fold (RR = 1.99, [1.62, 2.40]) (Fig 2B). Among metabolic comorbidities, there were moderate mortality risk increases associated with DM (RR = 1.67, [1.49, 1.86]) and obesity (RR = 1.24, [1.11, 1.37]) (Fig 2C). Patients with CKD had an increased risk of mortality (RR = 1.78, [1.55,2.03]) (Fig 2D), though it is important to note that the stage of CKD was not indicated in the MSHS database. Overall, the risk of mortality was approximately 2-fold greater in individuals with at least one chronic comorbidity in any category (p < 0.0001) (Fig 2A–2D).

The prevalence (absolute number and percentage) of individuals with each comorbidity within their indicated ethnic group is summarized in Table 2.

**Table 2:** Prevalence of comorbidities by ethnicities.

	Race and Ethnicity						
	White	Hispanic	Black or African American	Other	Asian	Native Hawaiian or Pacific Islander	American Indian or Alaskan Native
**Comorbidity**	**Absolute Number (%)**						
Obesity	393 (21.9)	556 (29.3)	597 (30.6)	220 (23.1)	36 (11.3)	1 (16.7)	2 (66.7)
HTN	350 (19.5)	543 (28.6)	502 (25.8)	201 (21.1)	83 (26.1)	1 (16.7)	1 (33.3)
DM	174 (9.7)	359 (18.9)	333 (17.1)	143 (15.0)	57 (17.9)	-	1 (33.3)
CAD	178 (9.9)	177 (9.3)	132 (6.8)	70 (7.3)	35 (11.0)	-	-
CKD	85 (4.7)	178 (9.4)	214 (11.0)	51 (5.4)	30 (9.4)	-	-
HF	96 (5.4)	99 (5.2)	114 (5.8)	24 (2.5)	15 (4.7)	1 (16.7)	-
Afib	121 (6.7)	73 (3.9)	64 (3.3)	29 (3.0)	12 (3.8)	-	-
Asthma	45 (2.5)	104 (5.5)	96 (4.9)	39 (4.1)	3 (0.9)	1 (16.7)	-
COPD	50 (2.8)	53 (2.8)	61 (3.1)	14 (1.5)	4 (1.3)	-	-

Distribution of COVID-19 positive patients in the Mount Sinai Health System by race and ethnicity and specific comorbidities is shown. HTN, hypertension; DM, diabetes mellitus; CAD, coronary artery disease; CKD, chronic kidney disease; HF, heart failure; Afib, atrial fibrillation; COPD, chronic obstructive pulmonary disease.

### Sequelae of COVID-19 and mortality

We evaluated the risk of mortality in COVID-19 patients who developed a clinical complication, i.e., sequelae of COVID-19. Overall, 476 individuals were reported to have developed at least one sequela. The percentages of the CoV2+ population with at least one developed sequela that were included in our analyses are 4.5% renal, 1.6% cardiovascular, 0.6% cerebrovascular, and 0.4% pulmonary (Table 3A). The prevalence of specific sequelae in descending order includes—acute kidney injury (AKI), acute myocardial infarction (AMI), acute venous thromboembolism (AVTE), cerebrovascular infarction (CVA), acute respiratory distress syndrome (ARDS), and intracerebral hemorrhage (ICH) (Table 3B).

**Table 3:** Overall prevalence of COVID-19 sequela.

**A.**	
**Sequela Categories**	**Percent**
Renal	4.5
Cardiovascular	1.6
Cerebrovascular	0.6
Pulmonary	0.4
**B.**	
**Sequela**	**Absolute Number (%)**
Acute Kidney Injury (AKI)	309 (4.5)
Acute Myocardial Infarction (AMI)	68 (1.0)
Acute Venous Thromboembolism (AVTE)	39 (0.6)
Cerebral Infarction (CVA)	37 (0.5)
Acute Respiratory Distress Syndrome (ARDS)	28 (0.4)
Intracerebral Hemorrhage (ICH)	7 (0.1)

(A) Of the 7,032 patients who presented to the Mount Sinai Health System with confirmed or presumed COVID-19 disease, we assessed the prevalence and associated risk of mortality in individuals who developed at least one renal, cardiovascular, cerebrovascular, or pulmonary complication secondary to COVID-19 disease. (B) The breakdown of prevalence by all specific sequelae considered is shown. Of note, patients may be represented in more than one group based on the number of complications they developed.

**Fig 3:**
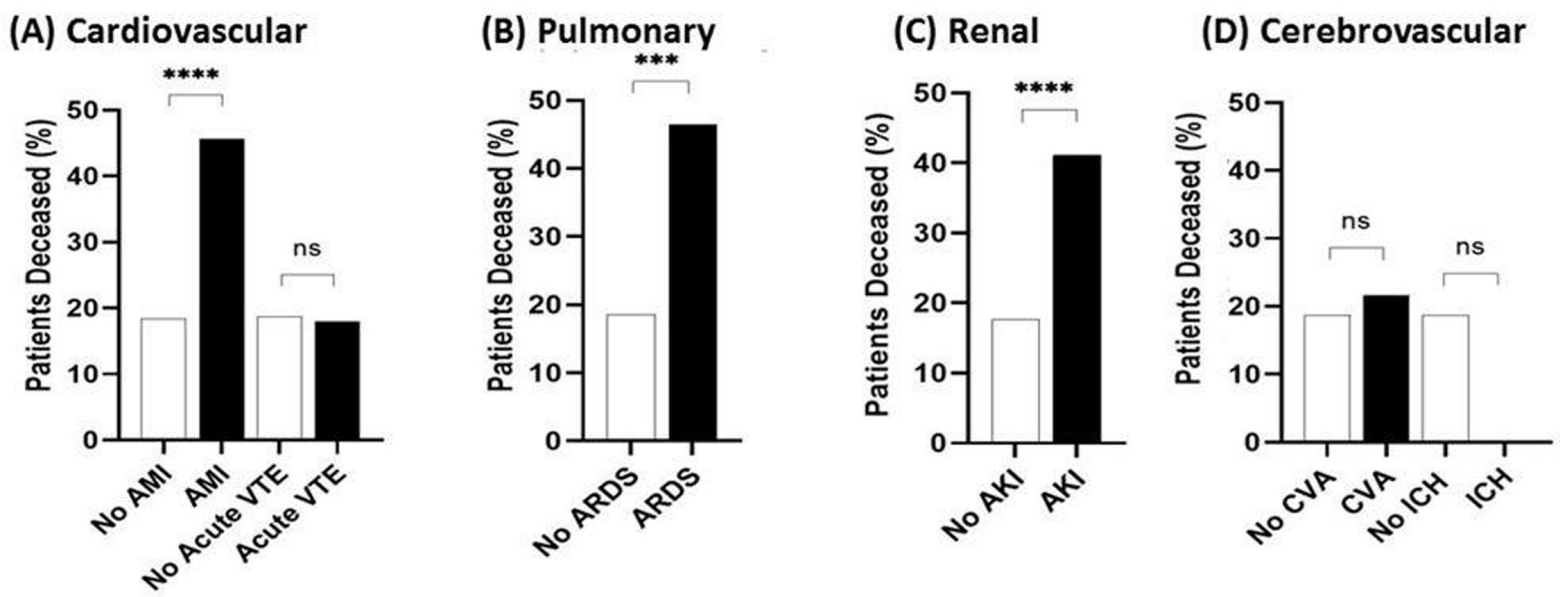
Relative risk of mortality in individuals who developed a COVID-19 sequela across all ages, sexes, and ethnicities. Of the 7,032 patients who presented to the Mount Sinai Health System with confirmed or presumed COVID-19 disease and known discharge/deceased status, we assessed the risk of mortality in individuals who developed at least one complication secondary to COVID-19 disease, with a particular focus on (A) cardiovascular, (B) pulmonary, (C) renal, and (D) cerebrovascular sequelae. Risk of mortality was greater among all populations which had developed acute kidney injury (AKI) or acute respiratory distress syndrome (ARDS). With regards to cardiovascular sequelae, patients who developed acute myocardial injury (AMI) had a 2.523-fold increased relative risk of mortality. In contrast, risk of mortality was not significant among a population with documented acute venous thromboembolism (acute VTE, p>0.9999). Relative risk of mortality was no significant among individuals who developed cerebral infarcts (CVA, p = 0.67) or intracranial hemorrhages (ICH, p = 0.36). ****p < 0.0001, ***p <0.001, **p <0.01, *p <0.05, ns = not significant.

The mortality risks are elevated in all populations that developed an AMI (RR = 2.47, [1.85, 3.13]), ARDS (RR = 2.50, [1.59, 3.47]), and AKI (RR = 2.33,[2.01, 2.67]), but not AVTE (RR = 0.96, [0.48, 1.79]), CVA (RR = 1.157, [0.61,2.00]), or ICH (RR = 0.00, [0.00, 1.89]) (Fig 3A–3D).

We further risk-stratified patients based on their ethnicity and sequelae. The prevalence (absolute number and percentage) of individuals who developed each specific sequela within their indicated ethnic group showed that clinical complications secondary to COVID-19 appeared to have poorer outcomes and were not associated with patient’s ethnicity (Table 4).

**Table 4:** Prevalence of sequela by ethnicity.

	Race and Ethnicity				
	White	Hispanic	Black or African American	Other	Asian
Sequela	Absolute Number (%)				
Acute Kidney Injury (AKI)	70 (3.9)	65 (3.4)	107 (5.5)	55 (5.8)	12 (3.8)
Acute Myocardial Infarction (AMI)	20 (1.1)	16 (0.8)	16 (0.8)	9 (0.9)	7 (2.2)
Acute Venous Thromboembolism (AVTE)	8 (0.5)	13 (0.7)	13 (0.7)	3 (0.3)	2 (0.6)
Cerebral Infarction (CVA)	4 (0.2)	11 (0.6)	15 (0.8)	6 (0.6)	1 (0.3)
Acute Respiratory Distress Syndrome (ARDS)	10 (0.6)	5 (0.3)	5 (0.3)	5 (0.5)	3 (0.9)
Intracerebral Hemorrhage (ICH)	1 (0.1)	1 (0.1)	5 (0.3)	-	-

Distribution of COVID-19 patients in the Mount Sinai Health System by ethnicity and COVID-19 sequelae is shown.

### Serum biomarkers of inflammation, coagulation, and cardiac injury

**Fig 4:**
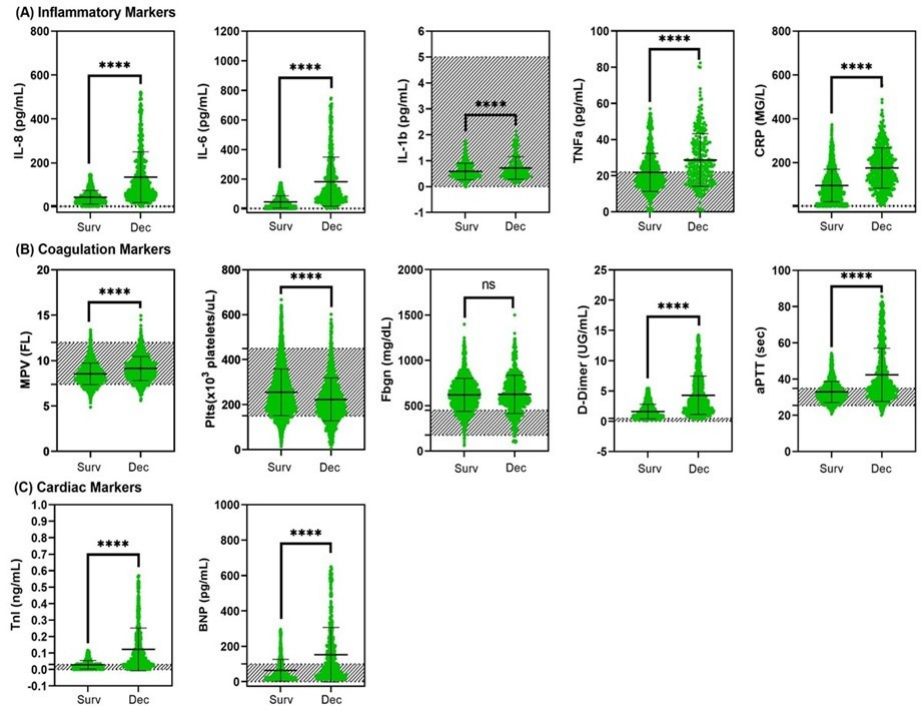
Mean comparison of peripheral blood biomarkers of cardiac injury, coagulation, and inflammation in surviving COVID-19 patients compared to deceased individuals. Within the 7,032 patients who presented to the Mount Sinai Health System with confirmed or presumed SARS-CoV2 infection and known discharge/deceased status, we compared–(A) the mean of inflammation markers [IL-8, IL-6, IL-1b, TNFα, C reactive protein (CRP)], (B) the mean of coagulation markers [mean platelet volume (MPV), platelets (Plts), fibrinogen (fbgn), d-dimer, and activated partial prothrombin time (aPTT)], and (C) the mean cardiovascular biomarkers [troponin (TnI) and brain natriuretic peptide (BNP)], between surviving and deceased individuals. For all individuals with multiple lab values were recorded into the database, the average of all values measured for a given biomarker was used in our analysis. Shaded areas reflect normal reference ranges for respective lab values. ****p < 0.0001, ***p <0.001, **p <0.01, *p <0.05, ns = not significant.

We assessed whether established inflammatory, coagulation, cardiovascular clinical biomarkers could provide further insight into risk of mortality in CoV2+ individuals (Fig 4A–4C and Table 5). All values for a given biomarker from a single patient are averaged if more than one result is available. The mean across patients for that biomarker is then calculated within the survivors and deceased patients for comparison. The mean ± standard deviation, the prevalence of patients outside the reference range, and the number of subjects with at least one laboratory test value for a given biomarker are provided in Table 5. Of note, the presence of large deviation in reported values can possibly be attributed to significant inter-patient variability in the context of where in the course of disease the patient is, disease severity, as well as response to treatment.

**Table 5:** Serum biomarkers of cardiac injury, coagulation, and inflammation in surviving COVID-19 patients compared to deceased individuals.

		Survivors		Deceased		P-value*
	Reference Range	Average ± STD	N	Average ± STD	N	
***Inflammatory Markers***						
IL-8 (pg/mL)	0–5	43.6 ± 30.9	1,416	134.8 ± 115.3	566	<0.0001
IL-6 (pg/mL)	0–5	47.3 ±41.3	2,089	183.9 ±165.9	650	<0.0001
IL-1b (pg/mL)	0–5	0.6 ± 0.3	564	0.72 ±0.4	211	<0.0001
TNFα (pg/mL)	0–5	21.8 ± 10.6	832	28.6 ± 14.6	280	<0.0001
CRP (mg/L)	0–5	95.3 ± 74.0	1,683	175.5 ± 92.2	569	<0.0001
***Coagulation Markers***						
MPV (FL)	7.4–12.0	8.5 ± 1.2	3,854	9.1 ± 1.3	1,253	<0.0001
Plts (x10^3^ platelets/uL)	150–450 x 10^3^	255 ± 104	3,920	223 ± 96	1,258	0.54
Fibrinogen (mg/dL)	175–450	619 ± 181	1,483	625 ± 211	627	<0.0001
D-dimer (ug/mL)	0–0.5	1.6 ± 1.21	4,290	4.3 ±3.2	1,160	<0.0001
aPTT (sec)	25.4–34.9	32.9 ± 5.9	1,732	42.3 ± 14.7	731	<0.0001
***Cardiac Biomarkers***						
Troponin I (ng/mL)	0–0.03	0.03 ± 0.1	1,823	0.1 ± 0.1	975	<0.0001
BNP (pg/mL)	0–100	63.8 ± 62.2	1,181	152.3 ± 154.4	651	<0.0001

Within the 7,032 patients who presented to the Mount Sinai Health System with confirmed or presumed COVID-19 disease and known discharge/deceased status, we compared the mean of cardiac, cardiovascular biomarkers [troponin (TnI) and brain natriuretic peptide (BNP)], the mean of markers of inflammation [IL-8, IL-6, IL-1b, TNFα, C reactive protein (CRP)] as well as the mean of markers of coagulation [mean platelet volume (MPV), platelets (Plts), fibrinogen, d-dimer, and activated partial prothrombin time (aPTT)] between surviving and deceased individuals. For all individuals with multiple lab values recorded into the database, the average of all values measured for a given biomarker was used in our analysis. The mean value for each biomarker (average ± STD) across all survivors, the number of survivors included in our analysis with at least one lab value for the given biomarker are listed here. The same is done for deceased individuals.

#### Inflammatory biomarkers and mortality

The levels of several inflammatory biomarkers were elevated in CoV2+ patients, and the degree by which these markers were elevated appears to be associated with poorer clinical outcomes. The levels of IL-8, IL6, and CRP were elevated above the normal range in both survived and deceased patients; however, all three inflammatory markers were approximately 2-4-fold higher in deceased COVID-19 patients compared to patients who recovered (p<0.0001) (Fig 4A and Table 5). Although the deceased patients had a small 20% increase (p<0.0001) in IL-1β levels compared to the survived patient population, these levels remained within the normal reference range for IL-1β (0–5 pg/mL) in both patient populations (Fig 4A and Table 5). The average levels of TNFα were increased by approximately 30% (p<0.0001) in the deceased patients compared to survivors. In both patient populations, these increases were above the normal range (0–22 pg/mL) of serum TNFα (Fig 4A and Table 5). It is important to note that levels of interleukins, TNFα, and CRP were tested almost exclusively in hospitalized COVID-19 patients. Thus our inflammatory biomarkers data represent COVID-19 patients with moderate-severe disease, but not asymptomatic or mild cases.

#### Coagulation markers and mortality

Since the beginning of the COVID-19 pandemic, significant evidence had emerged for increased incidence of coagulopathy, endotheliopathy, and various thrombotic and bleeding events in patients with COVID-19 [17–22]. We evaluated the levels of several coagulation markers in our CoV2+ patient population to elucidate underlying patterns (Fig 4B). The average mean platelet volume (MPV) levels were mostly within the reference range for both survivors and deceased patients. However, there was a very small 7% increase in MPV levels for deceased patients compared to survivors (Fig 4B and Table 5). Platelet (Plt) count remained within the lower threshold of normal; however, deceased patients had 12.5% (p<0.0001) lower average Plt count compared to survivors (Fig 4B and Table 5). The average levels of fibrinogen were increased by approximately 50% in both survivors and deceased patients, approaching the levels (>700 mg/dL) that can promote the formation of clots. D-dimer was elevated above the upper limit of normal in both survived and deceased patients; however, the average d-dimer levels were more than 250% (p<0.0001) higher in the deceased population compared to survivors (Fig 4B and Table 5). The increased levels of fibrinogen and d-dimer above normal thresholds in both groups may suggest an increased VTE risk with presence of macro- and/or micro-thrombi. These findings could suggest that peak values of these markers may be associated with a worse clinical outcome and the need for early administration of anticoagulant therapy as indicated by Paranjpe et al. [23]. Average activated partial prothrombin time (aPTT) values were prolonged by approximately 30% (p<0.0001) in deceased patients compared to those who survived (Fig 4B and Table 5). Taken together, dysregulation of coagulation markers can significantly affect the clotting cascade, and coagulopathies must be considered as one of the major sequelae of COVID-19, considering the reported incidence of thrombi as well as clotting factor deficiencies noted in other studies [23, 24].

#### Cardiovascular markers and mortality

While average cardiac troponin-I (cTnI) levels were at the upper limit of normal in the majority of COVID-19 patients that survived, there was more than a 330% (p<0.0001) increase in average cTnI levels in deceased patients (Fig 4C and Table 5), suggesting that the extent of myocardial injury is associated with worse clinical outcomes. Brain natriuretic peptide (BNP) levels were also significantly increased by more than 200% (p<0.0001) in deceased patients compared to survivors (Fig 4C and Table 5). Although cardiac structural and functional studies are unavailable in this de-identified database study, the substantially elevated levels of cardiovascular biomarkers in deceased patients suggest that significant myocardial injury could predict poorer outcomes in COVID-19 patients.

## Discussion

Since the initial reports of COVID-19 in early December 2019, the novel coronavirus outbreak continues to strain the global population, while its pathogenesis and clinical outcomes are not fully understood, and therapies continue to evolve. Using de-identified data from one of the largest US patient cohorts across a range of clinical services including outpatient (OP), emergency department (ED), inpatient (IP), and intensive care unit (ICU) admission, we assessed the associations between various demographic factors including age, sex, ethnicity on SARS-CoV2 qRT-PCR testing and COVID-19 clinical course severity and mortality. Of 39,539 patients who entered the MSHS with a COVID-19 concern as of June 8^th^ 2020, 7,032 patients (17.9%) were positive or presumed positive for COVID-19 of which 1,295 patients passed away (18.4%) while 5,737 recovered and were discharged (81.6%). Within this confirmed CoV2+ population, we evaluated a large panel of peripheral blood biomarkers, the impact of several comorbidities, and COVID-19 sequelae on patient outcomes.

Multiple epidemiological studies have reported that cardiovascular comorbidities are associated with COVID-19 disease severity and poorer outcomes [25–28]. Within our CoV2+ patient population, there was a high prevalence of cardiovascular and metabolic comorbidities compared to renal and pulmonary comorbidities. Despite difference in prevalence, comorbidities in these four major tested categories were associated with increased mortality risk, which is consistent with previous reports [12, 29–31]. Asthma was the only comorbidity that was not associated with increased mortality risk. Lack of associated mortality risk in patients with asthma may suggest that these individuals have some type of "hypoxic preconditioning" effect due to the occurrence of repetitive acute hypoxic episodes during exacerbations of their disease, a phenomenon recently described in a murine model [32]. In addition, it is also conceivable that chronic management of asthma attacks with inhaled corticosteroids may also provide an enhanced protective effect as a substantial number of patients administered steroids in combination with other therapies had shown improved outcomes in the setting of COVID-19 [33].

Regardless of ethnicities, patients with underlying HTN, CAD, HF, AFib, DM, obesity, COPD, and CKD experienced a higher risk of mortality compared to patients without these comorbidities. Indeed, the presence of at least one cardiovascular, renal, or pulmonary comorbidity increased the probability of poorer outcomes. However, we could not find significant race/ethnicity-associated modifications of mortality risks for most COVID-19 patients with these comorbidities, except for obesity, for which the risk of mortality was unchanged in the Hispanic population. These findings emphasize that in all ethnic groups, these comorbidities pose risks for worse clinical outcomes for COVID-19 patients, as reported before for other patient cohorts nationally and internationally [12, 29–31]. Of note, in spite of small differences observed between ethnic groups in terms of the degree by which comorbidity affects mortality rates, our analysis is limited to comparing whether patients have established comorbidity and does not take into consideration the patient’s age, stage of disease, or the therapy they have been receiving for their underlying medical condition(s).

Analysis of the prevalence of COVID-19 complications/sequelae revealed that renal and cardiovascular complications were the most prevalent, followed by cerebrovascular and pulmonary. Across all ethnic groups, the highest mortality risk was found in patients that developed AKI, followed by AMI and ARDS. While ethnicity-associated modifications were observed for AMI and ARDS, the AKI risk was not modified by ethnicity. All ethnic groups, except Black patients with AMI, were at increased risk of mortality due to COVID-19. Further analysis of clinical data, which was not available in the MSHS de-identified dataset, would be required to understand the potential causes of these findings. Although, the Black COVID-19 patients had the lowest prevalence for developing ARDS, we found that ARDS was associated with the highest risk of mortality in the Black population which is consistent with prior published studies [34, 35]. The worse outcomes due to ARDS in this population may be attributed to a more severe disease course or underlying comorbidities, which could increase predisposition for the development of ARDS.

Surprisingly, AVTE events and CVAs were not associated with an increased risk of mortality, potentially due to the administration of anticoagulation therapy in most of these patients. This may be attributed to early initiation of venous thromboembolism prophylaxis as of April 10^th^ 2020, with low molecular weight heparin (LMWH) in hospitalized patients in the MSHS with suspected or qRT-PCR confirmed COVID-19 [36].

In our analyses of peripheral blood biomarkers for cardiovascular disease, coagulation, and inflammation, elevation of these markers was consistently higher in the deceased versus recovered COVID-19 patients. Most of these circulating markers were above upper threshold, but IL-1β remained within normal limits for both patient populations. However, even these IL-1β values were statistically higher in deceased patients. A prior study of cytokines in a limited COVID-19 cohort study of 40 ICU patients reported IL-1β levels to be elevated above the upper limit of normal [37]. Considering our sample size and IL-1β’s role in the initiation of cytokine release syndrome and induced secretion of IL-6 [38], it may be advisable to consider the ratio of these cytokines (i.e. IL-1β/IL-6) to better understand the cytokine profile in COVID-19 patients and the associated inflammatory state.

CRP, IL-6, IL-8, and TNFα levels were significantly higher in deceased patients, suggesting a greater degree of systemic inflammation perpetuated by a "cytokine storm" which is supported by significant elevations in IL-6, IL-8, and TNFα [2, 7, 39, 40]. Overall, these findings reflect more active systemic inflammation in moderate/severe cases, which may contribute to the development of secondary sequelae, including renal failure or acute liver injury, supporting the concept of a high-risk inflammatory state secondary to COVID-19 and the associated higher risk of mortality.

Coagulation markers, including d-dimer and fibrinogen levels, were significantly elevated above the upper limit of normal in both patient groups suggesting COVID-19 is associated with coagulopathies [41]. Deceased patients had higher circulating d-dimer and fibrinogen compared to surviving patients, which may be reflective of the extent of micro- and macro-thrombi formation observed in disseminated intravascular coagulation [42]. An earlier study from NYU also showed 86% of their patients (n = 2,782) had elevated d-dimers (>230 ng/mL) with mean peak of d-dimer at 767 ng/ml, and those with elevated d-dimers were more likely to become critically ill [43]. Thus, our findings support the use of d-dimer as a biomarker for clinical course severity as well as being associated with poor outcomes.

Cardiac injury and stress are consistent with cardiac troponin and BNP elevation in the COVID-19 patient population and could serve as potential predictors for the development of COVID-19 sequelae such as AMI, AKI, HF exacerbation, or pulmonary edema. In our study, the COVID-19 deceased population had 3-fold higher mean cTnI levels compared to survivors. Although there are many potential etiologies of myocardial injury in the setting of COVID-19, including myocarditis [44], septic and/or stress cardiomyopathy [45, 46], or secondary hypoxic injury [47], our study did show that elevation in cTnI may be associated with the degree of multisystem organ dysfunction, and thus disease severity. These findings are consistent with an earlier report from the MSHS, where even small amounts of myocardial injury (cTnI > 0.03 to 0.09 ng/ml) were associated with higher risk of mortality [48]. Earlier reports from Wuhan, China also demonstrated elevated troponin in hospitalized patients with levels of greater than 2.14 mg/L predicting in-hospital mortality [49]. Within our patient population, the distribution of BNP also varied between survivors and deceased individuals. Considering the lack of further clinical data, elevation in BNP may also be multifactorial with either intrinsic cardiac dysfunction or secondary etiologies such as pulmonary edema in the setting of acute respiratory distress and renal disease. Earlier studies have also shown elevated cTnI [50] and BNP [51] in patients with COVID-19. However, given the variance in assays and lack of details regarding their upper thresholds, direct comparisons cannot be made for cTnI and BNP values in our studies and those of others. Despite these limitations, our data supports the presence of cardiovascular involvement in COVID-19, and more importantly reiterates the predictive clinical valve of cTnI and BNP for disease severity and outcomes.

In summary, our study is utilizing one of the most diverse and largest COVID-19 patient databases to study various factors that can influence susceptibility to infection and clinical outcomes. We found that—*i)* underlying metabolic and cardiovascular comorbidities are most prevalent in our COVID-19 patient population and were associated with increased mortality risk along with COPD and CKD; *ii)* asthma did not modify mortality risk in CoV2+ patients; *iii)* development of AKI, AMI and ARDS secondary to COVID-19 was associated with increased risk of mortality; *iv)* d-dimer, CRP, IL-6, TNFα, cTnI, and BNP levels were significantly elevated in deceased individuals. Taken together with previously published results, the finding in our study should assist clinicians and other health care workers in devising strategies to mitigate the risk of infection, assist with determining the timing for initiation of given therapies as well as altering them to optimize medical management.

### Study limitations

There are limitations in our study worth highlighting. Although, we included hospice patients in our relative risk analysis, we included hospice patients that were not indicated as deceased in the recovered category. However, this represents a very small portion of survivors or recovered patients (0.48%). Representing them in such a way introduces only a 2.12% reduction in deceased patients. Our analysis also included all emergency department and outpatients. However, their outcomes are not known unless they returned to MSHS for follow-up. Similarly, on discharge, inpatients could have been lost to follow-up, so their outcomes are not known unless they returned to MSHS. While other studies have used hazard functions to address this issue, they have focused on hospitalized populations. Our study facilitates assessment for looking at risks in the overall population entering the health system(s). Risks may still be overstated as many patients remain asymptomatic or have mild symptoms and therefore may not enter hospital systems. The false-negative rate of qRT-PCR tests for CoV2 infection is also a limitation in all current population reports, with studies estimate the sensitivity of most tests to be 70% [52].

## Abbreviations

Afib: atrial fibrillation
AKI: acute kidney injury
AMI: acute myocardial injury
aPTT: activated partial prothrombin time
ARDS: acute respiratory distress syndrome
AVTE: acute venous thromboembolism
BNP: brain natriuretic peptide
CAD: coronary artery disease
CKD: chronic kidney disease
COPD: chronic obstructive pulmonary disease
COVID-19: coronavirus disease 2019
CRP: C reactive protein
CVA: cerebrovascular infarction
DM: diabetes mellitus
Fbgn: fibrinogen
HF: Heart failure
HTN: hypertension
ICH: intracerebral hemorrhage
IL-1b: interleukin 1 beta
IL-6: interleukin 6
IL-8: interleukin 8
LMWH: low molecular weight heparin
MPV: mean platelet volume
Plts: platelets
RR: relative risk
SARS-CoV2: Severe acute respiratory syndrome coronavirus 2
TNFa: tumor necrosis factor alpha
TnI: troponin I

## References

[1] Chen N, Zhou M, Dong X, Qu J, Gong F, Han Y, et al. Epidemiological and clinical characteristics of 99 cases of 2019 novel coronavirus pneumonia in Wuhan, China: a descriptive study. Lancet. 2020;395(10223):507–13. Epub 2020/02/03. doi: 10.1016/S0140-6736(20)30211-7 ; PubMed Central PMCID: PMC7135076. 32007143 PMC7135076

[2] Zhou F, Yu T, Du R, Fan G, Liu Y, Liu Z, et al. Clinical course and risk factors for mortality of adult inpatients with COVID-19 in Wuhan, China: a retrospective cohort study. Lancet. 2020;395(10229):1054–62. Epub 2020/03/15. doi: 10.1016/S0140-6736(20)30566-3 ; PubMed Central PMCID: PMC7270627. 32171076 PMC7270627

[3] Xu Z, Shi L, Wang Y, Zhang J, Huang L, Zhang C, et al. Pathological findings of COVID-19 associated with acute respiratory distress syndrome. Lancet Respir Med. 2020;8(4):420–2. Epub 2020/02/23. doi: 10.1016/S2213-2600(20)30076-X ; PubMed Central PMCID: PMC7164771. 32085846 PMC7164771

[4] Guo T, Fan Y, Chen M, Wu X, Zhang L, He T, et al. Cardiovascular Implications of Fatal Outcomes of Patients With Coronavirus Disease 2019 (COVID-19). JAMA Cardiol. 2020. Epub 2020/03/29. doi: 10.1001/jamacardio.2020.1017 ; PubMed Central PMCID: PMC7101506. 32219356 PMC7101506

[5] Shi S, Qin M, Shen B, Cai Y, Liu T, Yang F, et al. Association of Cardiac Injury With Mortality in Hospitalized Patients With COVID-19 in Wuhan, China. JAMA Cardiol. 2020. Epub 2020/03/27. doi: 10.1001/jamacardio.2020.0950 ; PubMed Central PMCID: PMC7097841. 32211816 PMC7097841

[6] Wang D, Hu B, Hu C, Zhu F, Liu X, Zhang J, et al. Clinical Characteristics of 138 Hospitalized Patients With 2019 Novel Coronavirus-Infected Pneumonia in Wuhan, China. JAMA. 2020. Epub 2020/02/08. doi: 10.1001/jama.2020.1585 ; PubMed Central PMCID: PMC7042881. 32031570 PMC7042881

[7] Yang X, Yu Y, Xu J, Shu H, Xia J, Liu H, et al. Clinical course and outcomes of critically ill patients with SARS-CoV-2 pneumonia in Wuhan, China: a single-centered, retrospective, observational study. Lancet Respir Med. 2020;8(5):475–81. Epub 2020/02/28. doi: 10.1016/S2213-2600(20)30079-5 ; PubMed Central PMCID: PMC7102538. 32105632 PMC7102538

[8] Guan WJ, Ni ZY, Hu Y, Liang WH, Ou CQ, He JX, et al. Clinical Characteristics of Coronavirus Disease 2019 in China. N Engl J Med. 2020;382(18):1708–20. Epub 2020/02/29. doi: 10.1056/NEJMoa2002032 ; PubMed Central PMCID: PMC7092819. 32109013 PMC7092819

[9] Huang C, Wang Y, Li X, Ren L, Zhao J, Hu Y, et al. Clinical features of patients infected with 2019 novel coronavirus in Wuhan, China. Lancet. 2020;395(10223):497–506. Epub 2020/01/28. doi: 10.1016/S0140-6736(20)30183-5 ; PubMed Central PMCID: PMC7159299. 31986264 PMC7159299

[10] Liu H, Chen S, Liu M, Nie H, Lu H. Comorbid Chronic Diseases are Strongly Correlated with Disease Severity among COVID-19 Patients: A Systematic Review and Meta-Analysis. Aging Dis. 2020;11(3):668–78. Epub 2020/06/04. doi: 10.14336/AD.2020.0502 ; PubMed Central PMCID: PMC7220287. 32489711 PMC7220287

[11] Espinosa OA, Zanetti ADS, Antunes EF, Longhi FG, Matos TA, Battaglini PF. Prevalence of comorbidities in patients and mortality cases affected by SARS-CoV2: a systematic review and meta-analysis. Rev Inst Med Trop Sao Paulo. 2020;62:e43. Epub 2020/06/25. doi: 10.1590/S1678-9946202062043 ; PubMed Central PMCID: PMC7310609. 32578683 PMC7310609

[12] Wang T, Du Z, Zhu F, Cao Z, An Y, Gao Y, et al. Comorbidities and multi-organ injuries in the treatment of COVID-19. Lancet. 2020;395(10228):e52. Epub 2020/03/15. doi: 10.1016/S0140-6736(20)30558-4 ; PubMed Central PMCID: PMC7270177. 32171074 PMC7270177

[13] Gupta A, Madhavan MV, Sehgal K, Nair N, Mahajan S, Sehrawat TS, et al. Extrapulmonary manifestations of COVID-19. Nat Med. 2020;26(7):1017–32. Epub 2020/07/12. doi: 10.1038/s41591-020-0968-3 . 32651579 PMC11972613

[14] Kermali M, Khalsa RK, Pillai K, Ismail Z, Harky A. The role of biomarkers in diagnosis of COVID-19—A systematic review. Life Sci. 2020;254:117788. Epub 2020/06/02. doi: 10.1016/j.lfs.2020.117788 ; PubMed Central PMCID: PMC7219356. 32475810 PMC7219356

[15] Henry BM, de Oliveira MHS, Benoit S, Plebani M, Lippi G. Hematologic, biochemical and immune biomarker abnormalities associated with severe illness and mortality in coronavirus disease 2019 (COVID-19): a meta-analysis. Clin Chem Lab Med. 2020;58(7):1021–8. Epub 2020/04/15. doi: 10.1515/cclm-2020-0369 . 32286245

[16] Ponti G, Maccaferri M, Ruini C, Tomasi A, Ozben T. Biomarkers associated with COVID-19 disease progression. Crit Rev Clin Lab Sci. 2020;57(6):389–99. Epub 2020/06/07. doi: 10.1080/10408363.2020.1770685 ; PubMed Central PMCID: PMC7284147. 32503382 PMC7284147

[17] McFadyen JD, Stevens H, Peter K. The Emerging Threat of (Micro)Thrombosis in COVID-19 and Its Therapeutic Implications. Circ Res. 2020;127(4):571–87. Epub 2020/06/27. doi: 10.1161/CIRCRESAHA.120.317447 ; PubMed Central PMCID: PMC7386875. 32586214 PMC7386875

[18] Connors JM, Levy JH. COVID-19 and its implications for thrombosis and anticoagulation. Blood. 2020. Epub 2020/04/28. doi: 10.1182/blood.2020006000 . 32339221 PMC7273827

[19] McBane RD 2nd, Torres Roldan VD, Niven AS, Pruthi RK, Franco PM, Linderbaum JA, et al. Anticoagulation in COVID-19: A Systematic Review, Meta-analysis, and Rapid Guidance From Mayo Clinic. Mayo Clin Proc. 2020;95(11):2467–86. Epub 2020/11/07. doi: 10.1016/j.mayocp.2020.08.030 ; PubMed Central PMCID: PMC7458092. 33153635 PMC7458092

[20] Voicu S, Delrue M, Chousterman BG, Stepanian A, Bonnin P, Malissin I, et al. Imbalance between procoagulant factors and natural coagulation inhibitors contributes to hypercoagulability in the critically ill COVID-19 patient: clinical implications. Eur Rev Med Pharmacol Sci. 2020;24(17):9161–8. Epub 2020/09/24. doi: 10.26355/eurrev_202009_22866 . 32965009

[21] Manolis AS, Manolis TA, Manolis AA, Papatheou D, Melita H. COVID-19 Infection: Viral Macro- and Micro-Vascular Coagulopathy and Thromboembolism/Prophylactic and Therapeutic Management. J Cardiovasc Pharmacol Ther. 2021;26(1):12–24. Epub 2020/09/15. doi: 10.1177/1074248420958973 ; PubMed Central PMCID: PMC7492826. 32924567 PMC7492826

[22] Lodigiani C, Iapichino G, Carenzo L, Cecconi M, Ferrazzi P, Sebastian T, et al. Venous and arterial thromboembolic complications in COVID-19 patients admitted to an academic hospital in Milan, Italy. Thromb Res. 2020;191:9–14. Epub 2020/05/01. doi: 10.1016/j.thromres.2020.04.024 ; PubMed Central PMCID: PMC7177070. 32353746 PMC7177070

[23] Paranjpe I, Fuster V, Lala A, Russak A, Glicksberg BS, Levin MA, et al. Association of Treatment Dose Anticoagulation with In-Hospital Survival Among Hospitalized Patients with COVID-19. J Am Coll Cardiol. 2020. Epub 2020/05/11. doi: 10.1016/j.jacc.2020.05.001 . 32387623 PMC7202841

[24] Paranjpe I, Russak A, De Freitas JK, Lala A, Miotto R, Vaid A, et al. Clinical Characteristics of Hospitalized Covid-19 Patients in New York City. medRxiv: the preprint server for health sciences. 2020. Epub 2020/06/09. doi: 10.1101/2020.04.19.20062117 ; PubMed Central PMCID: PMC7277011. 32511655 PMC7277011

[25] Zhu L, She ZG, Cheng X, Qin JJ, Zhang XJ, Cai J, et al. Association of Blood Glucose Control and Outcomes in Patients with COVID-19 and Pre-existing Type 2 Diabetes. Cell metabolism. 2020;31(6):1068-77.e3. Epub 2020/05/06. doi: 10.1016/j.cmet.2020.04.021 ; PubMed Central PMCID: PMC7252168. 32369736 PMC7252168

[26] Zhao Q, Meng M, Kumar R, Wu Y, Huang J, Lian N, et al. The impact of COPD and smoking history on the severity of COVID-19: A systemic review and meta-analysis. Journal of medical virology. 2020. Epub 2020/04/16. doi: 10.1002/jmv.25889 ; PubMed Central PMCID: PMC7262275. 32293753 PMC7262275

[27] Sanyaolu A, Okorie C, Marinkovic A, Patidar R, Younis K, Desai P, et al. Comorbidity and its Impact on Patients with COVID-19. SN Compr Clin Med. 2020:1–8. Epub 2020/08/25. doi: 10.1007/s42399-020-00363-4 ; PubMed Central PMCID: PMC7314621. 32838147 PMC7314621

[28] Guan WJ, Liang WH, He JX, Zhong NS. Cardiovascular comorbidity and its impact on patients with COVID-19. The European respiratory journal. 2020;55(6). Epub 2020/04/29. doi: 10.1183/13993003.01227-2020 ; PubMed Central PMCID: PMC7236831. 32341104 PMC7236831

[29] Singh AK, Gupta R, Ghosh A, Misra A. Diabetes in COVID-19: Prevalence, pathophysiology, prognosis and practical considerations. Diabetes Metab Syndr. 2020;14(4):303–10. Epub 2020/04/17. doi: 10.1016/j.dsx.2020.04.004 ; PubMed Central PMCID: PMC7195120 interest related to this article. 32298981 PMC7195120

[30] Jain V, Yuan JM. Predictive symptoms and comorbidities for severe COVID-19 and intensive care unit admission: a systematic review and meta-analysis. Int J Public Health. 2020;65(5):533–46. Epub 2020/05/27. doi: 10.1007/s00038-020-01390-7 ; PubMed Central PMCID: PMC7246302. 32451563 PMC7246302

[31] Bansal M. Cardiovascular disease and COVID-19. Diabetes Metab Syndr. 2020;14(3):247–50. Epub 2020/04/05. doi: 10.1016/j.dsx.2020.03.013 ; PubMed Central PMCID: PMC7102662. 32247212 PMC7102662

[32] Setzke C, Pegelow DF, Broytman O, Teodorescu M. Effect of Repetitive Acute Hypoxic Preconditioning on Airway Reactivity During House Dust Mite—Induced Allergic Inflammation. A63 PATHOPHYSIOLOGY OF ASTHMA IN CELLS, TISSUES, AND ANIMAL MODELS. p. A2192-A.

[33] Group RC, Horby P, Lim WS, Emberson JR, Mafham M, Bell JL, et al. Dexamethasone in Hospitalized Patients with Covid-19—Preliminary Report. N Engl J Med. 2020. Epub 2020/07/18. doi: 10.1056/NEJMoa2021436 ; PubMed Central PMCID: PMC7383595. 32678530 PMC7383595

[34] Erickson SE, Shlipak MG, Martin GS, Wheeler AP, Ancukiewicz M, Matthay MA, et al. Racial and ethnic disparities in mortality from acute lung injury. Critical care medicine. 2009;37(1):1–6. Epub 2008/12/04. doi: 10.1097/CCM.0b013e31819292ea ; PubMed Central PMCID: PMC2696263. 19050621 PMC2696263

[35] Moss M, Mannino DM. Race and gender differences in acute respiratory distress syndrome deaths in the United States: an analysis of multiple-cause mortality data (1979–1996). Critical care medicine. 2002;30(8):1679–85. Epub 2002/08/07. doi: 10.1097/00003246-200208000-00001 . 12163776

[36] Wang Z, Zheutlin AB, Kao Y-H, Ayers KL, Gross SJ, Kovatch P, et al. Analysis of hospitalized COVID-19 patients in the Mount Sinai Health System using electronic medical records (EMR) reveals important prognostic factors for improved clinical outcomes. 2020:2020.04.28.20075788. doi: 10.1101/2020.04.28.20075788 J medRxiv

[37] McElvaney OJ, McEvoy NL, McElvaney OF, Carroll TP, Murphy MP, Dunlea DM, et al. Characterization of the Inflammatory Response to Severe COVID-19 Illness. Am J Respir Crit Care Med. 2020;202(6):812–21. Epub 2020/06/26. doi: 10.1164/rccm.202005-1583OC ; PubMed Central PMCID: PMC7491404. 32584597 PMC7491404

[38] Cavalli G, Dinarello CA. Anakinra Therapy for Non-cancer Inflammatory Diseases. Front Pharmacol. 2018;9:1157. Epub 2018/11/22. doi: 10.3389/fphar.2018.01157 ; PubMed Central PMCID: PMC6232613. 30459597 PMC6232613

[39] Rakhmanova AG, Prigozhina VK, Kunina TA. [Differential diagnosis of acute fatty liver and viral hepatitis in pregnancy]. Sov Med. 1988;(5):94–6. Epub 1988/01/01. . 3212542

[40] Manson JJ, Crooks C, Naja M, Ledlie A, Goulden B, Liddle T, et al. COVID-19-associated hyperinflammation and escalation of patient care: a retrospective longitudinal cohort study. Lancet Rheumatol. 2020;2(10):e594–e602. Epub 2020/08/31. doi: 10.1016/S2665-9913(20)30275-7 ; PubMed Central PMCID: PMC7442426. 32864628 PMC7442426

[41] Connors JM, Levy JH. COVID-19 and its implications for thrombosis and anticoagulation. Blood. 2020;135(23):2033–40. Epub 2020/04/28. doi: 10.1182/blood.2020006000 ; PubMed Central PMCID: PMC7273827. 32339221 PMC7273827

[42] Wada H, Hasegawa K, Watanabe M. DIC: an update on diagnosis and treatment. Rinsho Ketsueki. 2017;58(5):523–9. Epub 2017/06/09. doi: 10.11406/rinketsu.58.523 . 28592770

[43] Berger JS, Kunichoff D, Adhikari S, Ahuja T, Amoroso N, Aphinyanaphongs Y, et al. Prevalence and Outcomes of D-Dimer Elevation in Hospitalized Patients With COVID-19. Arterioscler Thromb Vasc Biol. 2020;40(10):2539–47. Epub 2020/08/26. doi: 10.1161/ATVBAHA.120.314872 ; PubMed Central PMCID: PMC7505147. 32840379 PMC7505147

[44] Kariyanna PT, Sutarjono B, Grewal E, Singh KP, Aurora L, Smith L, et al. A Systematic Review of COVID-19 and Myocarditis. Am J Med Case Rep. 2020;8(9):299–305. Epub 2020/08/05. ; PubMed Central PMCID: PMC7397751.32747875

[45] Singh S, Desai R, Gandhi Z, Fong HK, Doreswamy S, Desai V, et al. Takotsubo Syndrome in Patients with COVID-19: a Systematic Review of Published Cases. SN Compr Clin Med. 2020:1–7. Epub 2020/10/13. doi: 10.1007/s42399-020-00557-w ; PubMed Central PMCID: PMC7538054. 33043251 PMC7538054

[46] Arentz M, Yim E, Klaff L, Lokhandwala S, Riedo FX, Chong M, et al. Characteristics and Outcomes of 21 Critically Ill Patients With COVID-19 in Washington State. JAMA. 2020;323(16):1612–4. Epub 2020/03/20. doi: 10.1001/jama.2020.4326 ; PubMed Central PMCID: PMC7082763. 32191259 PMC7082763

[47] Creel-Bulos C, Hockstein M, Amin N, Melhem S, Truong A, Sharifpour M. Acute Cor Pulmonale in Critically Ill Patients with Covid-19. N Engl J Med. 2020;382(21):e70. Epub 2020/05/07. doi: 10.1056/NEJMc2010459 ; PubMed Central PMCID: PMC7281714. 32374956 PMC7281714

[48] Lala A, Johnson KW, Januzzi JL, Russak AJ, Paranjpe I, Richter F, et al. Prevalence and Impact of Myocardial Injury in Patients Hospitalized With COVID-19 Infection. J Am Coll Cardiol. 2020;76(5):533–46. Epub 2020/06/11. doi: 10.1016/j.jacc.2020.06.007 ; PubMed Central PMCID: PMC7279721. 32517963 PMC7279721

[49] Yao Y, Cao J, Wang Q, Shi Q, Liu K, Luo Z, et al. D-dimer as a biomarker for disease severity and mortality in COVID-19 patients: a case control study. J Intensive Care. 2020;8:49. Epub 2020/07/16. doi: 10.1186/s40560-020-00466-z ; PubMed Central PMCID: PMC7348129. 32665858 PMC7348129

[50] Kavsak PA, Hammarsten O, Worster A, Smith SW, Apple FS. Cardiac Troponin Testing in Patients with COVID-19: A Strategy for Testing and Reporting Results. Clin Chem. 2020. Epub 2020/10/13. doi: 10.1093/clinchem/hvaa225 ; PubMed Central PMCID: PMC7665403. 33045044 PMC7665403

[51] Andrew Abboud JLJ. Heart Failure Biomarkers in COVID-19. American College of Cardiology.

[52] Woloshin S, Patel N, Kesselheim AS. False Negative Tests for SARS-CoV-2 Infection—Challenges and Implications. N Engl J Med. 2020;383(6):e38. Epub 2020/06/06. doi: 10.1056/NEJMp2015897 . 32502334

